# Vitamin D Deficiency Is Associated with a Higher 5-Year Risk of Obstructive Sleep Apnea and CPAP Use in Older Adults: An Anchor-Based Network Meta-Analysis

**DOI:** 10.3390/medicina62050935

**Published:** 2026-05-11

**Authors:** Jui-Kun Chiang, Hsueh-Hsin Kao, Po-Han Chiang, Yee-Hsin Kao

**Affiliations:** 1 Department of Family Medicine, Dalin Tzu Chi Hospital, Buddhist Tzu Chi Medical Foundation, Chiayi 622, Taiwan; jkch68@gmail.com; 2 Department of Radiation Oncology, Taichung Veterans General Hospital, Taichung 407, Taiwan; kaogrady8176@gmail.com; 3 Great Tree Pharmacy (Dalin Branch), Chiayi 622, Taiwan; 4 Department of Family Medicine, Tainan Municipal Hospital (Managed by Show Chwan Medical Care Corporation), Tainan 701, Taiwan

**Keywords:** vitamin D, obstructive sleep apnea (OSA), continuous positive airway pressure (CPAP)

## Abstract

*Background and Objectives*: Continuous positive airway pressure (CPAP) is the standard first-line treatment for patients with obstructive sleep apnea (OSA). Patients with OSA exhibit a higher prevalence of vitamin D deficiency, and CPAP treatment has been reported to improve vitamin D levels. Therefore, the aim of this study was to investigate the association between vitamin D deficiency and the risk of patients receiving a diagnosis of OSA or utilizing CPAP, using the TriNetX research network to obtain real-world data. *Materials and Methods*: A retrospective cohort study using the TriNetX database was conducted to investigate the relationship between vitamin D deficiency and patients with risk of receiving an OSA diagnosis or patients treated with CPAP in older adults (≥65 years). Patients were stratified into four groups according to serum 25-hydroxyvitamin D [25(OH)D] levels: severe deficiency (D10, ≤10 ng/mL), mild deficiency (D20, 11–20 ng/mL), insufficient vitamin D status (S30, 21–30 ng/mL), and normal vitamin D status (N100, 31–100 ng/mL). After 1:1 propensity score matching (PSM) to adjust for baseline covariates, patients were followed for up to 5 years for newly diagnosed OSA and CPAP use (an administrative-clinical outcome influenced by non-disease-related factors such as healthcare access and clinical practice), with vitamin D status assessed at the index date. An anchor-based network meta-analysis was also performed to integrate direct and indirect evidence across groups. *Results*: A total of 2,289,855 eligible patients were included and stratified into four groups: D10 (*n* = 161,610; 7.06%), D20 (*n* = 404,954; 17.68%), S30 (*n* = 648,989; 28.34%), and N100 (*n* = 1,074,302; 46.92%). Across the six pairwise comparisons, pre-matching baseline imbalances showed consistent patterns: lower vitamin D groups (particularly D10 and D20) generally had older age at index (in comparisons with S30 or D20), a higher proportion of males, and higher proportions of Black/African American patients, whereas higher vitamin D groups (especially N100 and S30) had higher proportions of White patients (and, in some comparisons, Asian patients). Comorbidity differences were modest overall, and these imbalances were substantially reduced after PSM. In both pairwise PSM analyses and the anchor-based network meta-analysis, severe vitamin D deficiency was observationally associated with the risk of receiving an OSA diagnosis and CPAP use. An observational trend appeared across vitamin D categories (D10 > D20 > S30), suggesting an association. The associations were strongest at 1 year and gradually attenuated over the 3- and 5-year follow-up periods. An E-value greater than 3 was observed only for the comparison between the D10 and N100 groups. *Conclusions*: In this real-world analysis of older adults, vitamin D deficiency, particularly severe deficiency, was observationally associated with increased 5-year risk of receiving an OSA diagnosis and CPAP use. Similarly, anchor-based network meta-analysis also showed an association between vitamin D deficiency severity and the risk of receiving an OSA diagnosis and CPAP use.

## 1. Introduction

Obstructive sleep apnea (OSA) is a severe sleep disorder that may deteriorate the quality of life and lead to hypertension and cardiovascular and cerebrovascular diseases [1]. A previous systematic review reported that severe OSA was associated with a range of comorbidities, including heart disease, stroke, kidney disease, asthma, chronic obstructive pulmonary disease, acute and chronic heart failure, hyperlipidemia, thyroid disorders, cerebral infarction or embolism, myocardial infarction, and psychological conditions such as stress and depression [2]. Multiple risk factors for OSA have been identified, including advancing age; body mass index (BMI) ≥ 25 kg/m^2^; alcohol use; cigarette smoking; male sex; and increased neck circumference [3]. Anatomical contributors include upper-airway narrowing from tonsillar hypertrophy and reduced pharyngeal muscle tone in the posterior tonsillar region [2]. Ethnicity also appears to modulate risk; for example, individuals of Chinese descent commonly exhibit craniofacial features-such as a narrower cranial base (shorter thyromental distance) and a flatter midface (larger thyromental angle)-that influence airway collapsibility [4]. In addition, endocrine and metabolic disorders have been implicated, and emerging evidence suggests that vitamin D deficiency may be associated with an increased risk of receiving an OSA diagnosis. Continuous Positive Airway Pressure (CPAP) is the standard first-line treatment for patients with OSA [5].

A previous systematic review demonstrated an independent association between vitamin D status and OSA, showing that patients with OSA have lower serum 25(OH)D levels and a higher prevalence of vitamin D deficiency than controls, independent of age and BMI [6]. Consistently, a meta-analysis reported that serum 25(OH)D levels are not significantly reduced in patients with mild OSA, whereas individuals with moderate to severe OSA exhibit lower serum 25(OH)D concentrations than controls [7]. Taken together, these findings indicate that serum vitamin D levels decrease as OSA severity increases [8,9,10,11]. A previous review suggested a potential association between OSA and reduced vitamin D levels and indicated that CPAP therapy may have beneficial effects on vitamin D status [12]. Subsequent studies have shown that CPAP treatment can improve vitamin D levels, possibly by alleviating nocturnal hypoxia [13,14,15]. Furthermore, one study reported that serum vitamin D levels increased after 12 months of CPAP therapy, and that patients with good CPAP adherence exhibited higher serum 25(OH)D concentrations than those with poor adherence [11].

The primary function of vitamin D is the regulation of bone homeostasis [16]. However, vitamin D has also been implicated in several non-skeletal conditions, including cardiovascular disease, cancer, autoimmune disorders, and diabetes mellitus [17]. In recent years, the frequency of vitamin D testing has increased exponentially [18]. Vitamin D status is commonly assessed by measuring serum 25-hydroxyvitamin D [25(OH)D] levels. Concentrations below 20 ng/mL are considered deficient, levels between 20 and 30 ng/mL indicate insufficiency, and levels ≥30 ng/mL are regarded as sufficient [19]. Using these criteria, it is estimated that approximately 1 billion people worldwide have vitamin D deficiency or insufficiency [20]. Vitamin D deficiency remains highly prevalent globally; a recent report found a pooled prevalence of 47.9% for serum 25-hydroxyvitamin D [25(OH)D] levels <20 ng/mL during 2000–2022. This substantial burden represents a major public health challenge and contributes to the global burden of disease [21]. Vitamin D deficiency is associated with increased autoimmunity and susceptibility to infection [22]. Vitamin D receptors have been identified in multiple brain regions, including the hypothalamus, which plays a key role in regulating the sleep–wake cycle. Vitamin D deficiency shares several common risk factors with OSA, including older age, obesity, hypertension, chronic kidney disease, and diabetes mellitus [9,23,24]. OSA has also been linked to reduced 25(OH)D levels, as this sleep disorder leads to sleep fragmentation and daytime sleepiness, which may increase the risk of vitamin D deficiency [9,25].

Global populations are aging rapidly: by 2030, one in six people will be aged ≥60 years, with the ≥60 population projected to increase from 1.0 billion (2020) to 1.4 billion (2030) and 2.1 billion (2050), while those aged ≥80 years are expected to triple to 426 million by 2050 [26]. Aging is accompanied by substantial changes in sleep parameters and an increase in sleep complaints; accordingly, OSA is highly prevalent in older adults, affecting 25–46% in population-based studies [27]. Prior cohort studies further suggest that, in both men and women, OSA prevalence among adults aged 65–100 years is approximately twice that observed in middle age [28,29]. Vitamin D deficiency is common among community-dwelling older adults in higher-latitude developed countries and especially prevalent in institutionalized and frail elderly (e.g., geriatric and hip-fracture patients) [30]. It is a well-established risk factor for osteoporosis, falls, and fractures. A previous U.S. study reported that approximately 24% of adults aged ≥60 years have vitamin D deficiency, defined as a serum 25-hydroxyvitamin D [25(OH)D] concentration <20 ng/mL [31].

Although a previous study reported that sustained vitamin D deficiency was independently associated with an increased risk of OSA, the temporal consistency and pattern of associations may suggest a possible link [32]. In that study, serum 25(OH)D concentrations were dichotomized into a deficiency group (≤20 ng/mL) and a sufficiency group (≥30 ng/mL). In the current study, to more precisely characterize the associations, we stratified patients into four categories according to 25(OH)D level: severe deficiency (≤ 10 ng/mL; D10), mild deficiency (11–20 ng/mL; D20), insufficient (21–30 ng/mL; S30), and normal (31–100 ng/mL; N100). We aimed to investigate the association between serum vitamin D levels (across these four groups) and OSA, or treated with CPAP in older adults, using real-world data obtained from the TriNetX research network.

## 2. Materials and Methods

### 2.1. Data Sources and Ethical Approval

This study used data from the TriNetX Network (Cambridge, MA, USA), an international collaborative health research platform that aggregates de-identified patient data from electronic health records (EHRs) of participating healthcare organizations. The database includes comprehensive patient information, including demographics, clinical diagnoses (coded using ICD-10-CM), medical procedures (classified by ICD-10-PCS or Current Procedural Terminology [CPT]), laboratory tests (coded with Logical Observation Identifiers Names and Codes [LOINC]), and healthcare utilization records [33]. The TriNetX database undergoes rigorous data preprocessing and quality control to minimize missing values and ensure data standardization within a consistent framework. Further details about the database have been described elsewhere [34]. Because the TriNetX database contains only anonymized data, written informed consent was not required. The study protocol was reviewed and approved by the Institutional Review Board of the Research Ethics Committee of Buddhist Dalin Tzu Chi Hospital, Taiwan (No. B11402057).

### 2.2. Definitions of Variables in the Current Study

In the present study, patients were categorized into four groups according to serum 25-hydroxyvitamin D [25(OH)D] concentrations: severe deficiency (≤10 ng/mL; D10), mild deficiency (11–20 ng/mL; D20), insufficient (21–30 ng/mL; S30), and normal (31–100 ng/mL; N100) [20,30,35,36,37]. Outcomes were then compared across groups using anchor-based network meta-analysis.

OSA and CPAP: In the current study, older adults (aged ≥65 years) with OSA were identified using the ICD-10-CM diagnosis code G47.33. The CPAP cohort was defined as patients with evidence of CPAP use (an administrative-clinical outcome influenced by non-disease-related factors such as healthcare access and clinical practice), identified by procedure codes for CPAP initiation and management (CPT 94660; SNOMED CT 47545007; ICD-10-PCS 5A09357) in conjunction with an OSA diagnosis.

Type 2 diabetes mellitus (T2D), hypertension, overweight and obesity, hyperparathyroidism, chronic kidney disease (CKD) and liver cirrhosis: Previous studies have reported that parathyroid disorders, chronic kidney disease (CKD), and chronic liver disease play a crucial role in determining serum vitamin D levels [38,39]. In the current study, comorbidities were identified using ICD-10-CM diagnosis codes: hyperparathyroidism (E21), T2D (E11), hypertension (I10), overweight and obesity (E66), chronic kidney disease (N18), and liver cirrhosis (K74).

### 2.3. Index Date and Data Collection Process

The date of the first serum 25-hydroxyvitamin D measurement was defined as the index date. Each two groups were subsequently followed for 1, 3, and 5 years to evaluate clinical outcomes. OSA was identified using the ICD-10-CM code G47.33, and CPAP therapy was identified using procedure codes for CPAP initiation and management (UMLS: CPT:94660; ICD-10-PCS:5A09357). We then compared the incidence and outcomes of OSA and CPAP treatment between the four groups.

### 2.4. Inclusion and Exclusion Criteria

We included participants aged ≥65 years who had a recorded serum 25-hydroxyvitamin D measurement between 1 January 2013, and 30 November 2025. Participants were classified into four groups according to serum 25-hydroxyvitamin D [25(OH)D] concentrations: severe deficiency (≤10 ng/mL; D10), mild deficiency (11–20 ng/mL; D20), insufficient (21–30 ng/mL; S30), and normal (31–100 ng/mL; N100). Patients were excluded if they had any pre-existing sleep disorder at baseline-insomnia (ICD-10: G47.0), hypersomnia (G47.1), circadian rhythm sleep disorders (G47.2), or sleep apnea (G47.3), or conditions predisposing to sleep-disordered breathing, including congenital malformation syndromes (Q87), congenital skull/facial bone malformations (Q75), cleft lip/palate (Q35–Q37), neuromuscular disorders (G70-G73), and advanced kidney disease (end-stage renal disease [N18.6] or CKD stage 4–5 [N18.4–N18.5]). Additional exclusions were acromegaly/pituitary gigantism (E22.0), disorders of the nose and nasal sinuses (J34), and malignant neoplasms of the head, face, and neck (C76.0).

### 2.5. Matching Variables

These matching variables included demographic factors (age at index, gender, and race); comorbidities [overweight and obesity, type 2 diabetes, hypertension, hyperparathyroidism, chronic kidney disease, and cirrhosis].

### 2.6. Outcome Measurement and Follow-Up Period

The outcomes of this study were risks of receiving an OSA diagnosis and the initiation of CPAP treatment, assessed at 1, 3, and 5 years following the index date or until the date of data analysis on 27 November 2025.

### 2.7. Statistical Analysis

Statistical analyses were conducted using the integrated functions of the TriNetX platform. Baseline characteristics of the continuous values were reported as means and standard deviations, and categorial data were presented as counts with percentages. Welch modified two-sample *t*-test was used to compare the continuous data. The chi-squared test or Fisher’s exact test was used to compare the categorial items. To address imbalances in baseline covariates, 1:1 propensity score matching (PSM) was employed based on variables listed in the Methods section. The PSM used a nearest-neighbor matching algorithm with a caliper width of 0.1 pooled standard deviations. Variables with a standardized difference of less than 0.1 between groups were considered adequately balanced.

After matching, the cumulative incidence of each outcome was estimated using Cox regression models, with results expressed as hazard ratios (HRs) and 95% confidence intervals (CIs). Proportional hazard assumptions were also tested. Kaplan–Meier curves were generated to compare event-free distributions between groups, with statistical significance assessed using the log-rank test. These curves were plotted using R statistical software (version 4.5.2, R Foundation for Statistical Computing, Vienna, Austria) based on probabilities provided by TriNetX. A *p* value of <0.05 was considered statistically significant. Sensitivity analyses were performed to evaluate outcomes at 1, 3, and 5 years following the index date.

We further performed an anchor-based network meta-analysis using a common-comparator approach, in which the severe vitamin D deficiency group (≤10 ng/mL; D10) was designated as the anchor group to avoid double-counting participants from the same database [40]. We adopted its framework to integrate the direct and indirect evidence derived from our multiple pairwise matched cohorts within a frequentist approach. This allowed us to comprehensively evaluate all possible pairwise comparisons rather than relying on a single reference group. This approach represents an analytical comparison within a single database utilizing an anchor-based network meta-analysis framework to integrate evidence across matched cohorts, rather than a conventional meta-analysis of independent research studies.

Effect estimates were summarized as hazard ratios (HRs) with corresponding 95% confidence intervals (CIs). We applied either a random-effects model or a common-effect model, as appropriate, to account for potential heterogeneity across the different matched comparisons (rather than traditional between-study heterogeneity). Consistency between direct and indirect evidence was evaluated using the design-by-treatment interaction model. The different vitamin D strata were ranked using P-scores, with bigger values indicating a lower risk for the outcomes, further justifying the use of this framework to provide an intuitive hierarchy of the exposure categories. These analyses were performed in R using the netmeta 3.2.0 package. E-values were calculated with the Evalue 4.1.4 package.

## 3. Results

**Figure 1:**
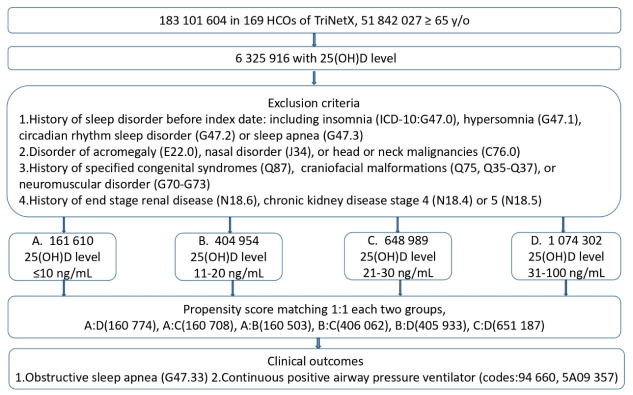
Flowchart of the patient selection process. Footnote: A represents D10, severe vitamin D deficiency (≤10 ng/mL); B represents D20, mild vitamin D deficiency (11–20 ng/mL); C represents S30, insufficient vitamin D status (21–30 ng/mL); and D represents N100, normal vitamin D status (31–100 ng/mL).

The flow chart of participant selection is shown in Figure 1. In the current study, 2,289,855 patients were categorized into four groups: D10 (*n* = 161,610; 7.06%), D20 (*n* = 404,954; 17.68%), S30 (*n* = 648,989; 28.34%), and N100 (*n* = 1,074,302; 46.92%). We subsequently performed pairwise matched comparisons to assess clinical outcomes between every two groups. As propensity score matching was performed separately for each pairwise analysis, the matched sample size of each group differed by comparison. Thus, slight variations in group numbers were observed across analyses.

In the current study, patients were categorized into four groups, resulting in six pairwise comparisons. Accordingly, the baseline characteristics of participants before and after propensity score matching were compared for each comparison. Given the space constraints and the overall organization of the manuscript, Table 1, Table 2 and Table 3 are presented in the main text, while the remaining tables are provided as Appendix A Table A1, Table A2 and Table A3.

Table 1 summarizes the baseline characteristics of patients in the severe vitamin D deficiency (D10) and normal vitamin D status (N100) groups before and after propensity score matching. Before matching, the D10 group had a higher proportion of males than the N100 group (34.4% vs. 28.4%; standardized difference [Std diff] = 0.130). Marked racial differences were observed: the N100 group had higher proportions of White patients (70.0% vs. 39.6%; Std diff = 0.642) and Asian patients (3.8% vs. 1.5%; Std diff = 0.142), whereas the D10 group had a higher proportion of Black/African American patients (15.1% vs. 7.6%; Std diff = 0.236). Regarding comorbidities, the D10 group had higher prevalence of diabetes mellitus (13.5% vs. 11.9%; Std diff = 0.048) and cirrhosis (1.4% vs. 0.8%; Std diff = 0.066), but a lower prevalence of hypertension (27.5% vs. 33.5%; Std diff = 0.130), than the N100 group.

Table 2 presents the baseline characteristics of patients in the mild vitamin D deficiency (D20) and insufficient vitamin D status (S30) groups before and after propensity score matching. Before matching, the D20 group had a higher proportion of males than the S30 group (36.0% vs. 33.3%; Std diff = 0.056). Racial differences were observed: the S30 group had a higher proportion of White patients (61.9% vs. 51.6%; Std diff = 0.210), whereas the D20 group had a higher proportion of Black/African American patients (12.8% vs. 8.9%; Std diff = 0.125). There were no significant differences between the D20 and S30 groups in the proportion of Asian patients or in comorbidities.

Table 3 presents the baseline characteristics of patients in the severe vitamin D deficiency (D10) and insufficient vitamin D status (S30) groups before and after propensity score matching. Before matching, the mean age at index was higher in the D10 group than in the S30 group (70.6 ± 10.7 years vs. 68.9 ± 9.6 years; Std diff = 0.166). Racial differences were also observed: the S30 group had higher proportions of White patients (61.9% vs. 39.6%; Std diff = 0.458) and Asian patients (3.5% vs. 1.5%; Std diff = 0.128), whereas the D10 group had a higher proportion of Black/African American patients (15.1% vs. 5.9%; Std diff = 0.190). Regarding comorbidities, the D10 group had a higher prevalence of cirrhosis (1.4% vs. 0.9%; Std diff = 0.053). The prevalence of hypertension also differed between groups (37.5% vs. 27.5%; Std diff = 0.087).

Appendix A Table A1, Table A2 and Table A3 present the baseline characteristics of patients in the D10 versus D20, D20 versus N100, and S30 versus N100 groups before and after propensity score matching. Before matching, patients in the D10 group were older than those in the D20 group, whereas patients in the N100 group were older than those in the D20 and S30 groups. With respect to sex distribution, the D20 and S30 groups had higher proportions of males than the N100 group. Racial differences were also observed across comparisons: the D20 and N100 groups generally included higher proportions of White and Asian patients, whereas the D10 and D20 groups included higher proportions of Black/African American patients. In addition, hypertension was less prevalent in the D10 group than in the D20 group and in the D20 group than in the N100 group, while diabetes mellitus was more prevalent in the D20 group than in the N100 group. Detailed data are provided in Appendix A Table A1, Table A2 and Table A3.

In summary, the mean age at index showed a consistent pattern before matching, with patients in the more severe deficiency groups (especially D10) and the normal group (N100) tending to be older than those in the D20 and S30 groups (e.g., D10 > S30 and D20; N100 > D20 and S30), and these age imbalances were attenuated after propensity score matching. The proportion of males was generally higher in the lower vitamin D groups, including D10 vs. N100, D20 vs. S30, D20 vs. N100, and S30 vs. N100 before matching, with improved balance after matching. Racial differences were consistently observed before matching: groups with higher vitamin D status (especially N100 and S30, and D20 vs. D10) tended to have higher proportions of White (and in several comparisons, Asian) patients, whereas groups with lower vitamin D status (particularly D10 and D20) tended to have higher proportions of Black/African American patients; these differences were reduced after matching. Regarding comorbidities, baseline differences were generally modest before matching, with lower vitamin D groups showing slightly higher prevalence of diabetes mellitus and cirrhosis in some comparisons (particularly D10 or D20 vs. N100) and lower prevalence of hypertension, while D20 vs. S30 and S30 vs. N100 showed no major comorbidity differences; these imbalances were largely mitigated after matching.

In pairwise propensity score-matched analyses, the severe deficiency (D10) group was observationally associated with a higher risk of receiving an OSA diagnosis than the other groups. The association was strongest for D10 versus N100, followed by D10 versus S30 and D10 versus D20 (HRs: 1.50, 1.22, and 1.08, respectively). A similar pattern was observed for CPAP use, with the strongest association for D10 versus N100, followed by D10 versus S30 and D10 versus D20 (HRs: 2.21, 1.88, and 1.33, respectively). Across pairwise comparisons, the associations for both OSA and CPAP use were strongest at 1 year and attenuated slightly at 3 and 5 years. For D10, the HRs for OSA were 1.50, 1.48, and 1.45 at 1, 3, and 5 years, respectively, and the corresponding HRs for CPAP use were 2.21, 1.94, and 1.85, respectively. In the pairwise propensity score-matched analysis, E-values exceeding 3 were observed only for the D10 versus N100 comparison across the 1-, 3-, and 5-year follow-up periods. (Table 4) E-values greater than 2 were observed for the comparisons of the D10 group vs. the N100 group for OSA, the D10 group vs. the S30 group for CPAP use, and the D20 group vs. the N100 group for both OSA and CPAP use. Moreover, we replaced obesity status with BMI in our primary analysis (Table 4) to generate Appendix A Table A4.

**Figure 2:**
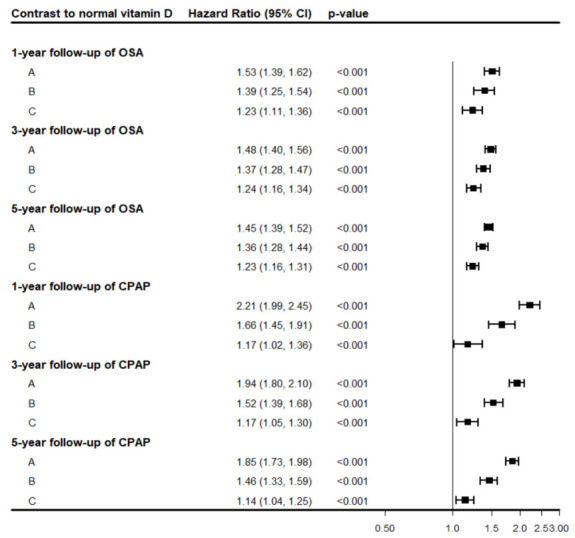
Anchor-based network meta-analysis of the risk of receiving an OSA diagnosis and CPAP use in the D10, D20, and S30 groups at 1, 3, and 5 years of follow-up, using the N100 group (normal vitamin D status, 31–100 ng/mL) as the reference. A, D10 (severe vitamin D deficiency, ≤10 ng/mL); B, D20 (mild vitamin D deficiency, 11–20 ng/mL); C, S30 (insufficient vitamin D status, 21–30 ng/mL).

Table 5 presents the anchor-based network meta-analysis of the risks of receiving an OSA diagnosis and CPAP use across serum vitamin D groups at 1-, 3-, and 5-year follow-up, with the normal group (N100) as the reference. We found that the strongest observational associations for receiving an OSA diagnosis and CPAP use were noted in the severe deficiency group (D10), followed by the mild deficiency group (D20) and the insufficiency group (S30). Across groups, the associations were strongest at 1 year, followed by 3 years and 5 years of follow-up. These findings support a possible association between the severity of vitamin D deficiency and the risks of receiving an OSA diagnosis and CPAP use. P-scores range from 0 to 1 and reflect the average certainty that one group ranks higher than competing groups, based on the magnitude and precision of the effect estimates. Higher P-scores indicate a higher ranking for the likelihood of receiving an OSA diagnosis or CPAP use. In the current study, the severe deficiency group (D10) had the highest P-score (1.0), followed by the D20 and S30 groups, for both OSA and CPAP use. Figure 2 presents the anchor-based network meta-analysis of the risks of receiving an OSA diagnosis and CPAP use in the D10, D20, and S30 groups at 1-, 3-, and 5-year follow-up, using the N100 group as the reference. In the pairwise propensity score-matched analysis, E-values exceeding 3 were observed only for the D10 versus N100 comparison across the 1-, 3-, and 5-year follow-up periods.

## 4. Discussion

In the current study, the severe vitamin D deficiency group (D10) was observationally associated with the risks of receiving an OSA diagnosis and CPAP use, followed by the mild deficiency group (D20) and the insufficient vitamin D group (S30) for older adults. Across groups, the associations were strongest at 1 year and attenuated over time at the 3- and 5-year follow-up assessments. One possible hypothesis-generating explanation is that patients with more severe vitamin D deficiency were more likely to receive vitamin D supplementation during follow-up once the deficiency was recognized. Nevertheless, the underlying mechanism remains uncertain and warrants further investigation in future prospective studies.

In the anchor-based network meta-analysis comparing serum vitamin D groups at 1, 3, and 5 years (with the normal group [N100] as the reference), the strongest association was consistently observed for D10 versus N100, followed by D10 versus S30 and D10 versus D20. Overall, these findings support a possible association between the severity of vitamin D deficiency and risks of receiving an OSA diagnosis and CPAP use. Only the D10 versus N100 comparison yielded an E-value greater than 3 for CPAP use across the 1-, 3-, and 5-year follow-up periods. However, E-values should be interpreted as supplementary statistical measures rather than definitive evidence of robustness. Even relatively high E-values cannot account for the absence of important clinical and behavioral variables in the database, including BMI, physical activity, smoking, alcohol use, sunlight exposure, vitamin D supplementation, and healthcare utilization. Accordingly, substantial residual confounding may still be present, and the observed association should be interpreted with caution rather than as evidence of robustness to unmeasured confounding. It is essential to recognize that the observed associations may partially reflect the patients’ overall health status and behavioral profiles. For instance, lower vitamin D levels could serve as a surrogate marker for frailty or reduced outdoor physical activity, which are also linked to OSA risk. These factors, alongside differences in diagnostic practices across healthcare systems, may contribute to the findings beyond a direct biological link.

In this real-world analysis using the TriNetX database, several baseline patient characteristics were also notable. Mean age at the index date followed a non-linear pattern, with patients in the more severe deficiency groups (particularly D10) and in the normal group (N100) tending to be older than those in the D20 and S30 groups. The proportion of male patients was generally higher in the lower vitamin D groups. Racial differences were also consistently observed, with higher vitamin D groups comprising larger proportions of White patients (and, in several comparisons, Asian patients), whereas lower vitamin D groups included larger proportions of Black/African American patients. With respect to comorbidities, lower vitamin D groups showed slightly higher prevalence of diabetes mellitus and cirrhosis in some comparisons, along with a modestly lower prevalence of hypertension.

These findings are broadly consistent with prior literature suggesting a bidirectional relationship between vitamin D status and OSA. A previous systematic review reported an association between moderate-to-severe OSA and low vitamin D levels, likely mediated by hypoxia-related pathways [25]. Moreover, sleep fragmentation and excessive daytime sleepiness, which are inherent features of OSA, may further increase the risk of vitamin D deficiency by limiting outdoor and other lifestyle activities [10]. In parallel, another study emphasized that consistent adherence to CPAP therapy remains essential for OSA management and for mitigating its complications [41]. A randomized sham-controlled trial further reported that CPAP may exert delayed beneficial effects and lead to greater improvements in vitamin D levels, particularly in patients with severe OSA [42]. In addition, a prospective study demonstrated that vitamin D levels improved in patients with OSA who received CPAP therapy, with more favorable outcomes observed among those with good CPAP adherence [11].

In the current study, severe vitamin D deficiency was observationally associated with higher CPAP use. However, this finding should be interpreted cautiously because CPAP use reflects an administrative–clinical pathway rather than a direct physiological measure of OSA severity. Such records may be influenced by access to sleep testing, physician prescribing patterns, insurance or coding practices, patient acceptance, and adherence-related factors. A previous systematic review reported that CPAP adherence rates have remained persistently low over the past two decades despite efforts to improve adherence through behavioral interventions and patient coaching [43]. From a biological perspective, previous studies have proposed several mechanisms linking vitamin D deficiency to OSA, including increased inflammation, oxidative stress, hypoxia, impaired immune and respiratory muscle function, and vitamin D-related genetic polymorphisms [44]. In addition, elevated heart rate, a surrogate marker of sympathetic nervous system activity, has been associated with lower 25-hydroxyvitamin D levels [8,45,46]. Although differences in CPAP adherence may also have contributed to the observed associations, adherence was not directly evaluated in the present study.

From a clinical perspective, our findings may support the importance of identifying and correcting true vitamin D deficiency in older adults, particularly in those at risk for OSA. According to a consensus statement from the 5th International Conference “Controversies in Vitamin D,” interventions to restore adequate vitamin D status are likely to be effective only in individuals who are truly deficient, regardless of the treatment strategy. Accordingly, treatment goals should focus on preventing serum 25(OH)D levels from falling below 12 ng/mL, with an optimal target of maintaining levels above 20 ng/mL [47]. Among the three major strategies for achieving vitamin D sufficiency-sunshine exposure, food fortification, and supplementation-supplementation may be the most practical and effective approach in aging populations [47]. Further prospective studies are warranted to determine whether vitamin D supplementation can improve OSA severity and reduce the need for CPAP use.

In the current study, the final analysis included more than 160,503 matched pairs of older adults across four vitamin D status groups, ranging from severe deficiency to normal status. This exceptionally large sample size represents a major strength of the study and provides statistical power for detecting the observed associations. Nevertheless, several limitations should be acknowledged. First, a prior literature-based analysis reported that, among 16 of 193 countries with available data, the prevalence of OSA was highest in China, followed by the United States, Brazil, and India. Using an apnea–hypopnea index threshold of ≥5 events per hour and the 2012 American Academy of Sleep Medicine criteria, that study estimated that approximately 936 million individuals aged 30–69 years worldwide have OSA [48]. However, a major limitation of the present study is that participants in the TriNetX database were predominantly from the United States, which may limit global generalizability. Race data depended on whether the participating institutions provided this information; when such data were unavailable, patients were categorized as having unknown race. Both the unknown race category and seasonal variation were assumed to be randomly distributed across the study population; therefore, their influence on the study results was expected to be limited. Second, the primary limitation of our study is its observational retrospective design, which precludes the establishment of a definitive causal relationship between vitamin D deficiency and risks of receiving an OSA diagnosis or CPAP use. As OSA often has a prolonged, undiagnosed preclinical phase, behavioral and physiological factors that might negatively influence vitamin D levels may already be present well before a formal diagnosis. Therefore, we cannot definitively assume that low vitamin D exposure reliably preceded the onset of OSA. Although statistically significant associations were observed, causality cannot be confirmed without randomized controlled trials or well-designed prospective interventional studies, and our findings must be interpreted strictly as associations. Third, our study is constrained by insufficient control of confounding factors due to the inherent limitations of the database. Although propensity score matching was applied to balance baseline characteristics and reduce confounding, residual confounding cannot be entirely excluded. Our matching procedure and multivariable models included only a limited set of variables. Several crucial determinants for both OSA and vitamin D status—including precise measures of obesity (e.g., neck circumference), lifestyle factors, dietary habits, seasonality, sun exposure, over-the-counter vitamin D supplementation, socioeconomic status, frailty, and detailed healthcare utilization metrics—were unavailable. Consequently, the risk of residual and systematic confounding remains possible. Although we calculated E-values to assess the potential impact of unmeasured confounding, this statistical approach cannot fully substitute for the actual measurement and adjustment of these critical clinical variables. Therefore, the E-values should not be interpreted as definitive evidence of robustness, and the associations observed may still be subject to unmeasured systematic biases. Fourth, we observed statistically significant associations between vitamin D deficiency and risk of receiving an OSA diagnosis or CPAP use. However, because OSA was identified solely through ICD codes, without polysomnographic confirmation or phenotypic characterization, the findings may reflect differences in diagnostic ascertainment and coding likelihood rather than true disease occurrence. In addition, given the large sample size, statistical significance does not necessarily indicate clinical significance. Therefore, the gap between statistical significance and clinical relevance should be regarded as an important limitation of the current study. Fifth, the use of CPAP as an outcome measure has important limitations. A record of CPAP use is not a direct biological or physiological indicator of OSA severity; rather, it reflects a complex clinical pathway influenced by multiple non-biological factors, including access to care, physician decision-making, coding practices, and patient adherence. Because our retrospective database lacks detailed polysomnographic information, CPAP use cannot be considered a reliable surrogate marker of severe OSA. Moreover, its dependence on factors unrelated to disease severity, such as nightly duration of use, should be explicitly acknowledged. Additionally, other non-disease-related factors may also impact CPAP use. Sixth, caution must be exercised when interpreting the gradient observed across the different vitamin D categories. Although we noted an association between lower vitamin D levels and an increased risk of receiving an OSA diagnosis, these findings are more appropriately interpreted as associations. The “normal” vitamin D group is inherently heterogeneous, and higher vitamin D levels may serve as a surrogate marker for better overall health status, healthier lifestyle behaviors, or greater outdoor physical activity. Seventh, the interpretation of our findings must account for the substantial sample size of our cohort. In large database studies, even trivial and clinically negligible differences can easily achieve statistical significance (e.g., *p* < 0.05) strictly due to the high statistical power. Therefore, the statistical significance observed in this study should not be conflated with clinical relevance. We urge readers to focus primarily on the magnitude of the effect sizes (the hazard ratios and odds ratios) and their corresponding confidence intervals, rather than solely on the *p*-values. Eighth, serum 25-hydroxyvitamin D [25(OH)D] levels were available only as group mean values, rather than individual-level data, which represents a limitation of the TriNetX database. Ninth, in the current study, the anchor-based network meta-analysis was based on a star-shaped network; therefore, all comparisons between non-reference groups relied solely on indirect evidence. Finally, patients included in the TriNetX electronic health record (EHR) database received care within 66 contributing healthcare systems, most of which are large academic medical centers. As a result, the generalizability of our findings to other clinical settings or populations should be validated using additional national or population-based data sources.

## 5. Conclusions

In this real-world analysis of more than 160,503 matched pairs of older adults across vitamin D status groups, severe vitamin D deficiency was observationally associated with an increased 5-year risk of receiving an OSA diagnosis and CPAP use. Similarly, anchor-based network meta-analysis also showed an association between vitamin D deficiency severity and risks of receiving an OSA diagnosis and CPAP use. The association between severe vitamin D deficiency and CPAP use was observed across the 1-, 3-, and 5-year follow-up periods. However, this finding should be interpreted cautiously, and the possibility of substantial residual confounding may remain. Prospective studies are warranted to determine whether vitamin D supplementation can improve OSA severity and reduce the need for CPAP use in older adults.

## Figures and Tables

**Table 1:** Baseline characteristics of the D10 (severe vitamin D deficiency) and N100 (normal vitamin D status) groups before and after propensity score matching.

Variable	Before Matching			After Matching		
	D10 Group (*n* = 161,241)	N100 Group (*n* = 1,083,634)	Std Diff	D10 Group (*n* = 160,774)	N100 Group (*n* = 160,774)	Std Diff
Mean age at index, years (SD)	70.6 (10.7)	70.1 (9.3)	0.044	70.6 (10.7)	70.5 (10.6)	0.004
Gender						
Female	105,437 (65.6)	774,468 (71.6)	0.129	105,437 (65.6)	105,407 (65.6)	<0.001
Male	55,346 (34.4)	307,327 (28.4)	0.130	55,318 (34.4)	55,299 (34.4)	<0.001
Race/ethnicity						
White	63,647 (39.6)	757,746 (70.0)	0.642	63,647 (39.6)	63,659 (39.6)	<0.001
Black or African American	24,225 (15.1)	82,663 (7.6)	0.236	24,225 (15.1)	24,484 (15.2)	0.004
Asian	2462 (1.5)	41,256 (3.8)	0.142	2462 (1.5)	2443 (1.5)	0.001
Unknown race	67,004 (41.7)	175,471 (16.2)	0.585	66,976 (41.7)	66,692 (41.5)	0.004
Other races	2807 (1.7)	18,889 (1.7)	<0.001	2807 (1.7)	2792 (1.7)	0.001
Comorbidities						
Hypertension	44,273 (27.5)	362,729 (33.5)	0.130	44,265 (27.5)	44,810 (27.9)	0.008
Type 2 diabetes	21,746 (13.5)	128,898 (11.9)	0.048	21,721 (13.5)	21,943 (13.6)	0.004
Hyperparathyroidism	2148 (1.3)	14,462 (1.3)	<0.001	2147 (1.3)	2001 (1.2)	0.008
Overweight and obesity	9434 (5.9)	62,450 (5.8)	0.004	9422 (5.9)	9640 (6.0)	0.006
Chronic kidney disease	9097 (5.7)	65,194 (6.0)	0.016	9096 (5.7)	9148 (5.7)	0.001
Liver cirrhosis	2304 (1.4)	8123 (0.8)	0.066	2276 (1.4)	2326 (1.4)	0.003

Abbreviation: D10, severe vitamin D deficiency (≤10 ng/mL); N100, normal vitamin D status (31–100 ng/mL).

**Table 2:** Baseline characteristics of the D20 (mild vitamin D deficiency) and S30 (insufficient vitamin D status) groups before and after propensity score matching.

Variable	Before Matching			After Matching		
	D20 Group (*n* = 407,625)	S30 Group (*n* = 653,594)	Std Diff	D20 Group (*n* = 406,062)	S30 Group (*n* = 406,062)	Std Diff
Mean age at index, years (SD)	68.7 (9.9)	68.9 (9.6)	0.017	68.7 (9.9)	68.8 (9.8)	0.004
Gender						
Female	260,121 (64.0)	434,795 (66.7)	0.055	260,066 (64.0)	260,663 (64.2)	0.003
Male	146,084 (36.0)	217,289 (33.3)	0.056	145,919 (35.9)	145,331 (35.8)	0.003
Race/ethnicity						
White	209,568 (51.6)	403,884 (61.9)	0.210	209,568 (51.6)	209,413 (51.6)	0.001
Black or African American	51,924 (12.8)	58,148 (8.9)	0.125	51,781 (12.8)	51,282 (12.6)	0.004
Asian	11,348 (2.8)	23,100 (3.5)	0.043	11,348 (2.8)	11,325 (2.8)	<0.001
Unknown race	121,929 (30.0)	149,483 (22.9)	0.161	121,853 (30.0)	122,502 (30.2)	0.003
Other races	8804 (2.2)	13,378 (2.1)	0.008	8803 (2.2)	8869 (2.2)	0.001
Comorbidities						
Hypertension	123,599 (30.4)	205,368 (31.5)	0.023	123,512 (30.4)	124,508 (30.7)	0.005
Type 2 diabetes	55,137 (13.6)	80,701 (12.4)	0.036	55,029 (13.6)	55,708 (13.7)	0.005
Hyperparathyroidism	4207 (1.0)	8323 (1.3)	0.023	4207 (1.0)	3876 (1.0)	0.008
Overweight and obesity	26,634 (6.6)	40,348 (6.2)	0.015	26,593 (6.5)	26,936 (6.6)	0.003
Chronic kidney disease	21,323 (5.2)	33,416 (5.1)	0.006	21,301 (5.2)	21,055 (5.2)	0.003
Liver cirrhosis	4951 (1.2)	5666 (0.9)	0.034	4834 (1.2)	4659 (1.1)	0.004

Abbreviation: D20, mild vitamin D deficiency (11–20 ng/mL); S30, insufficient vitamin D status (21–30 ng/mL).

**Table 3:** Baseline characteristics of the D10 (severe vitamin D deficiency) and S30 (insufficient vitamin D status) groups before and after propensity score matching.

Variable	Before Matching			After Matching		
	D10 Group (*n* = 161,241)	S30 Group (*n* = 635,595)	Std Diff	D10 Group (*n* = 160,708)	S30 Group (*n* = 160,708)	Std Diff
Mean age at index, years (SD)	70.6 (10.7)	68.9 (9.6)	0.166	70.6 (10.7)	70.5 (10.6)	0.004
Gender						
Female	105,437 (65.6)	434,795 (66.7)	0.023	105,393 (65.6)	105,575 (65.7)	0.002
Male	55,346 (34.4)	217,290 (33.3)	0.023	55,296 (34.4)	55,066 (34.3)	0.003
Race/ethnicity						
White	63,647 (39.6)	403,885 (61.9)	0.458	63,647 (39.6)	63,746 (39.7)	0.001
Black or African American	24,225 (15.1)	58,148 (5.9)	0.190	24,211 (15.1)	25,291 (15.7)	0.019
Asian	2462 (1.5)	23,100 (3.5)	0.128	2462 (1.5)	2446 (1.5)	0.001
Unknown race	67,004 (41.7)	149,483 (22.9)	0.409	66,924 (41.6)	65,762 (40.9)	0.015
Other races	2807 (1.7)	13,378 (2.1)	0.022	2807 (1.7)	2816 (1.8)	<0.001
Comorbidities						
Hypertension	44,273 (27.5)	205,369 (31.5)	0.087	44,261 (27.5)	44,976 (28.0)	0.010
Type 2 diabetes	21,746 (13.5)	80,702 (12.4)	0.034	21,668 (13.5)	22,310 (13.9)	0.012
Hyperparathyroidism	2148 (1.3)	8323 (1.3)	0.005	2140 (1.3)	1872 (1.2)	0.015
Overweight and obesity	9434 (5.9)	40,348 (6.2)	0.013	9417 (5.9)	9477 (5.9)	0.002
Chronic kidney disease	9097 (5.7)	33,416 (5.1)	0.024	9055 (5.6)	9339 (5.8)	0.008
Liver cirrhosis	2304 (1.4)	5666 (0.9)	0.053	2262 (1.4)	2371 (1.5)	0.006

Abbreviation: D10, severe vitamin D deficiency (≤10 ng/mL); S30, insufficient vitamin D status (21–30 ng/mL).

**Table 4:** Differences in clinical outcomes between the severe deficiency (D10) and normal (N100) groups across follow-up time points after propensity score matching.

Clinical Outcome (*n*)	Index Date to 1-Year Follow-Up				Index Date to 3-Year Follow-Up				Index Date to 5-Year Follow-Up			
	*n*	HR (95% CI)	*p*	*E*	*n*	HR (95% CI)	*p*	*E*	*n*	HR (95% CI)	*p*	*E*
D10 vs. N100 (160,774;160,774)	D10/ N100				D10/ N100				D10/ N100			
OSA	1642/ 1112	1.50 (1.39–1.62)	<0.001 (0.682)	2.37 (2.13)	3561/ 2494	1.48 (1.40–1.56)	<0.001 (0.279)	2.32 (2.15)	4924/ 3532	1.45 (1.39–1.52)	<0.001 (0.169)	2.26 (2.13)
CPAP use	1129/ 517	2.21 (1.99–2.45)	<0.001 (0.017)	3.85 (3.39)	1950/ 1032	1.94 (1.80–2.10)	<0.001 (0.002)	3.29 (3.0)	2594/ 1447	1.85 (1.74–1.98)	<0.001 (0.001)	3.10 (2.87)
D10 vs. S30 (160,708;160,708)	D10/ S30				D10/ S30				D10/ S30			
OSA	1642/ 1375	1.22 (1.14–1.31)	<0.001 (0.993)	1.74 (1.54)	3560/ 3135	1.19 (1.14–1.25)	<0.001 (0.271)	1.67 (1.54)	4923/ 4476	1.18 (1.13–1.23)	<0.001 (0.497)	1.64 (1.51)
CPAP use	1129/ 611	1.88 (1.70–2.08)	<0.001 (0.053)	3.17 (2.79)	1950/ 1225	1.66 (1.55–1.78)	<0.001 (0.010)	2.71 (2.47)	2594/ 1700	1.62 (1.52–1.72)	<0.001 (0.007)	2.62 (2.41)
D10 vs. D20 (160,503;160,503)	D10/ D20				D10/ D20				D10/ D20			
OSA	1641/ 1535	1.08 (1.01–1.16)	0.026 (0.192)	1.37 (1.11)	3560/ 3420	1.08 (1.03–1.13)	0.002 (0.365)	1.37 (1.21)	4923/ 4841	1.07 (1.03–1.11)	0.001 (0.515)	1.34 (1.21)
CPAP use	1129/ 854	1.33 (1.22–1.46)	<0.001 (0.427)	1.99 (1.74)	1949/ 1572	1.28(1.19–1.36)	<0.001 (0.827)	1.88 (1.67)	2592/ 2129	1.27 (1.20–1.35)	<0.001 (0.641)	1.86 (1.69)
D20 vs. S30 (406,062;406,062)	D20/ S30				D20/ S30				D20/ S30			
OSA	4493/ 4085	1.11 (1.07–1.16)	<0.001 (0.154)	1.46 (1.34)	10,005/ 9188	1.11 (1.08–1.14)	<0.001 (0.591)	1.46 (1.37)	14,212/ 13,116	1.11 (1.08–1.13)	<0.001 (0.602)	1.46 (1.37)
CPAP use	2374/ 1624	1.48 (1.39–1.57)	<0.001 (0.005)	2.32 (2.13)	4339/ 3237	1.36 (1.30–1.43)	<0.001 (<0.001)	2.06 (1.92)	5953/ 4569	1.33 (1.28–1.38)	<0.001 (<0.001)	1.99 (1.88)
D20 vs. N100 (405,933;405,933)	D20/ N100				D20/ N100				D20/ N100			
OSA	4495/ 3203	1.42 (1.35–1.48)	<0.001 (0.302)	2.19 (2.04)	10,007/ 7346	1.37 (1.33–1.42)	<0.001 (0.040)	2.08 (1.99)	14,213/ 10,334	1.37 (1.34–1.41)	<0.001 (0.360)	2.09 (2.01)
CPAP use	2373/ 1328	1.80 (1.68–1.92)	<0.001 (0.002)	3.00 (2.75)	4339/ 2726	1.60 (1.52–1.68)	<0.001 (<0.001)	2.58 (2.41)	5953/ 3858	1.53 (1.47–1.60)	<0.001 (<0.001)	2.43 (2.30)
S30 vs. N100 (651,187;651,187)	S30/ N100				S30/ N100				S30/ N100			
OSA	6705/ 5263	1.27 (1.23–1.32)	<0.001 (0.019)	1.86 (1.76)	15,310/ 12,085	1.25 (1.22–1.28)	<0.001 (0.368)	1.81 (1.74)	21,879/ 17,089	1.25 (1.23–1.28)	<0.001 (0.576)	1.81 (1.76)
CPAP use	2678/ 2154	1.24 (1.17–1.31)	<0.001 (0.116)	1.79 (1.62)	5327 4513	1.17 (1.12–1.21)	<0.001 (0.006)	1.62 (1.49)	7562/ 6390	1.15 (1.11–1.19)	<0.001 (0.093)	1.57 (1.46)

Abbreviation: CI, confidence interval; CPAP, continuous positive airway pressure; OSA, obstructive sleep apnea; D10, severe vitamin D deficiency (serum 25(OH)D concentration ≤10 ng/mL); D20, mild vitamin D deficiency (11–20 ng/mL); S30, insufficient vitamin D status (21–30 ng/mL); N100, normal vitamin D status (31–100 ng/mL); P(p) for log rank test (proportionality test); E-value (lower 95% C.I.).

**Table 5:** Anchor-based network meta-analysis of the risk of receiving an OSA diagnosis and CPAP use across serum vitamin D groups at 1-, 3-, and 5-year follow-up, with the normal group (N100) as the reference.

Clinical Outcome	Group	HR (95% C.I.)	*p* Value	P-Score
OSA, 1-year follow-up				
	D10	1.50 (1.39–1.62)	<0.001	0.995
	D20	1.39 (1.25–1.54)	<0.001	0.669
	S30	1.23 (1.11–1.36)	<0.001	0.336
	N100	Reference		0
OSA, 3-year follow-up				
	D10	1.48 (1.40–1.56)	<0.001	0.995
	D20	1.37 (1.28–1.47)	<0.001	0.669
	S30	1.24 (1.16–1.34)	<0.001	0.336
	N100	Reference		0
OSA, 5-year follow-up				
	D10	1.45 (1.39–1.52)	<0.001	0.995
	D20	1.36 (1.28–1.44)	<0.001	0.669
	S30	1.23 (1.16–1.31)	<0.001	0.336
	N100	Reference		0
CPAP use, 1-year follow-up				
	D10	2.21 (1.99–2.45)	<0.001	0.995
	D20	1.66 (1.45–1.91)	<0.001	0.669
	S30	1.17 (1.02–1.36)	<0.001	0.336
	N100	Reference		0
CPAP use, 3-year follow-up				
	D10	1.94 (1.80–2.10)	<0.001	0.995
	D20	1.52 (1.39–1.68)	<0.001	0.669
	S30	1.17 (1.05–1.30)	<0.001	0.336
	N100	Reference		0
CPAP use, 5-year follow-up				
	D10	1.85 (1.73–1.98)	<0.001	0.995
	D20	1.46 (1.33–1.59)	<0.001	0.669
	S30	1.14 (1.04–1.25)	<0.001	0.336
	N100	Reference		0

Abbreviation: CI, confidence interval; CPAP, continuous positive airway pressure; OSA, obstructive sleep apnea; D10, severe vitamin D deficiency (serum 25(OH)D concentration ≤10 ng/mL); D20, mild vitamin D deficiency (11–20 ng/mL); S30, insufficient vitamin D status (21–30 ng/mL); N100, normal vitamin D status (31–100 ng/mL). All heterogeneity tests were passed. (all *p* > 0.05).

## Data Availability

The datasets generated during and/or analyzed during the current study are not publicly available but are available from the corresponding author on reasonable request.

## Abbreviations

The following abbreviations are used in this manuscript:

BMI	Body Mass Index
CPAP	Continuous Positive Airway Pressure
D10	Severe vitamin D deficiency (serum 25(OH)D concentration ≤10 ng/mL)
D20	Mild vitamin D deficiency (11–20 ng/mL)
N100	Normal vitamin D status (31–100 ng/mL)
OSA	Obstructive Sleep Apnea
S30	Insufficient vitamin D status (21–30 ng/mL)
T2D	Type 2 Diabetes

## Appendix A

**Table A1:** Baseline characteristics of the D10 (severe vitamin D deficiency) and D20 (mild vitamin D deficiency) groups before and after propensity score matching.

Variable	Before Matching			After Matching		
	D10 Group (*n* = 161,241)	D20 Group (*n* = 404,625)	Std Diff	D10 Group (*n* = 160,503)	D20 Group (*n* = 160,503)	Std Diff
Mean age at index, years (SD)	70.6 (10.7)	68.7 (9.9)	0.179	70.6 (10.7)	70.6 (10.6)	0.001
Gender						
Female	105,437 (65.6)	260,121 (64.0)	0.032	105,200 (65.5)	105,350 (65.6)	0.002
Male	55,346 (34.4)	146,084 (36.0)	0.032	55,287 (34.4)	55,096 (34.3)	0.003
Race/ethnicity						
White	63,647 (39.6)	209,568 (51.6)	0.243	63,647 (39.7)	63,681 (39.7)	<0.001
Black or African American	24,225 (15.1)	51,924 (12.8)	0.066	24,208 (15.1)	24,605 (15.3)	0.007
Asian	2462 (1.5)	11,348 (2.8)	0.087	2462 (1.5)	2472 (1.5)	0.001
Unknown race	60,004 (41.7)	121,929 (30.0)	0.245	66,722 (41.6)	66,304 (41.3)	0.005
Other races	2807 (1.7)	8804 (1.7)	0.030	2807 (1.7)	2792 (1.7)	0.001
Comorbidities						
Hypertension	44,273 (27.5)	123,599 (30.4)	0.064	44,217 (27.5)	44,475 (27.7)	0.004
Type 2 diabetes	21,746 (13.5)	55,137 (13.6)	0.001	21,602 (13.5)	21,656 (13.5)	0.001
Hyperparathyroidism	2148 (1.3)	4207 (1.0)	0.028	2030 (1.3)	2028 (1.3)	<0.001
Overweight and obesity	9434 (5.9)	26,634 (6.6)	0.029	9409 (5.9)	9190 (5.7)	0.006
Chronic kidney disease	9097 (5.7)	21,323 (5.2)	0.018	8970 (5.6)	8931 (5.6)	0.001
Liver cirrhosis	2304 (1.4)	4951 (1.2)	0.019	2256 (1.4)	2232 (1.4)	0.003

Abbreviation: D10, severe vitamin D deficiency (≤10 ng/mL); D20, mild vitamin D deficiency (11–20 ng/mL).

**Table A2:** Baseline characteristics of the D20 (mild vitamin D deficiency) and N100 (normal vitamin D status) groups before and after propensity score matching.

Variable	Before Matching			After Matching		
	D20 Group (*n* = 407,625)	N100 Group (*n* = 1,083,634)	Std Diff	D20 Group (*n* = 405,933)	N100 Group (*n* = 405,933)	Std Diff
Mean age at index, years (SD)	68.7 (9.9)	70.1 (9.3)	0.145	68.7 (9.9)	68.8 (9.8)	0.008
Gender						
Female	260,121 (64.0)	774,468 (71.6)	0.162	260,108 (64.1)	260,413 (64.2)	0.002
Male	146,084 (36.0)	307,327 (28.4)	0.162	145,748 (35.9)	145,419 (35.8)	0.002
Race/ethnicity						
White	209,568 (51.6)	757,746 (70.0)	0.385	209,568 (51.6)	209,158 (51.5)	0.002
Black or African American	51,924 (12.8)	82,663 (7.6)	0.170	51,859 (12.8)	50,803 (12.5)	0.008
Asian	11,348 (2.8)	41,256 (3.8)	0.057	11,348 (2.8)	11,390 (2.8)	0.001
Unknown race	121,929 (30.0)	175,471 (16.2)	0.322	121,646 (30.0)	122,880 (30.3)	0.007
Other races	8804 (2.2)	18,889 (1.7)	0.030	8803 (2.2)	9082 (2.2)	0.005
Comorbidities						
Hypertension	123,599 (30.4)	362,729 (33.5)	0.066	123,506 (30.4)	125,260 (30.9)	0.009
Type 2 diabetes	55,137 (13.6)	128,898 (11.9)	0.050	55,006 (13.6)	56,561 (13.9)	0.011
Hyperparathyroidism	4207 (1.0)	14,462 (1.3)	0.028	4207 (1.0)	4039 (1.0)	0.004
Overweight and obesity	26,634 (6.6)	62,450 (5.8)	0.033	26,570 (6.5)	27,742 (6.8)	0.012
Chronic kidney disease	21,323 (5.2)	65,194 (6.0)	0.034	21,311 (5.2)	21,676 (5.3)	0.004
Liver cirrhosis	4951 (1.2)	8123 (0.8)	0.047	4859 (1.2)	4659 (1.1)	0.005

Abbreviation: D20, mild vitamin D deficiency (11–20 ng/mL); N100, normal vitamin D status (31–100 ng/mL).

**Table A3:** Baseline characteristics of the S30 (insufficient vitamin D status) and N100 (normal vitamin D status) groups before and after propensity score matching.

Variable	Before Matching			After Matching		
	S30 Group (*n* = 653,594)	N100 Group (*n* = 1,083,634)	Std Diff	S30 Group (*n* = 651,187)	N100 Group (*n* = 651,187)	Std Diff
Mean age at index, years (SD)	68.9 (9.6)	70.1 (9.3)	0.131	68.9 (9.6)	69.0 (9.5)	0.004
Gender						
Female	434,795 (66.7)	774,468 (71.3)	0.106	434,761 (66.8)	435,449 (66.9)	0.002
Male	217,289 (33.3)	307,327 (28.4)	0.107	216,255 (33.2)	215,573 (33.1)	0.002
Race/ethnicity						
White	403,884 (61.9)	757,746 (70.0)	0.172	403,884 (62.0)	403,033 (61.9)	0.003
Black or African American	58,148 (8.9)	82,663 (7.6)	0.046	58,133 (8.9)	57,946 (8.9)	0.001
Asian	23,100 (3.5)	41,256 (3.8)	0.014	23,100 (3.5)	23,006 (3.5)	0.001
Unknown race	149,483 (22.9)	175,471 (16.2)	0.170	148,430 (22.8)	149,644 (23.0)	0.004
Other races	13,378 (2.1)	18,889 (1.7)	0.022	13,378 (2.1)	13,395 (2.1)	<0.001
Comorbidities						
Hypertension	205,368 (31.5)	362,729 (33.5)	0.043	205,185 (31.5)	206,297 (31.7)	0.004
Type 2 diabetes	80,701 (12.4)	128,898 (11.9)	0.014	80,528 (12.4)	81,137 (12.5)	0.003
Hyperparathyroidism	8323 (1.3)	14,462 (1.3)	0.005	8311 (1.3)	8236 (1.3)	0.001
Overweight and obesity	40,348 (6.2)	62,450 (5.8)	0.018	40,275 (6.2)	40,378 (6.2)	0.001
Chronic kidney disease	33,416 (5.1)	65,194 (6.0)	0.039	33,404 (5.1)	33,394 (5.1)	<0.001
Liver cirrhosis	5666 (0.9)	8123 (0.9)	0.013	5647 (0.9)	5252 (0.8)	0.007

Abbreviation: S30, insufficient vitamin D status (21–30 ng/mL); and N100, normal vitamin D status (31–100 ng/mL).

**Table A4:** Differences in clinical outcomes between groups at each follow-up time point after propensity score matching (BMI instead of obesity).(D10, *n* = 156,304; D20, *n* = 404,954; S30, *n* = 606,670; N100, *n* = 1,003,559).

Clinical Outcome (*n*)	Index Date To 1-Year Follow-Up				Index Date to 3-Year Follow-Up				Index Date to 5-Year Follow-Up			
	*n*	HR (95% CI)	*p*	*E*	*n*	HR (95% CI)	*p*	*E*	*n*	HR (95% CI)	*p*	*E*
D10 vs. N100 (155,468 pairs)	D10/ N100				D10/ N100				D10/ N100			
OSA	1601/ 1071	1.52 (1.41–1.64)	<0.001 (0.332)	2.41 (2.17)	3478/ 2482	1.45 (1.38–1.53)	<0.001 (0.001)	2.26 (2.10)	4787/ 3467	1.44 (1.38–1.50)	<0.001 (0.023)	2.24 (2.10)
CPAP use	1109/ 514	2.18 (1.96–2.42)	<0.001 (0.002)	3.78 (3.33)	1931/ 1019	1.95 (1.81–2.10)	<0.001 (<0.001)	3.31 (3.02)	2532/ 1411	1.85 (1.74–1.98)	<0.001 (0.004)	3.10 (2.87)
D10 vs. S30 (155,518 pairs)	D10/ S30				D10/ S30				D10/ S30			
OSA	1602/ 1375	1.19 (1.11–1.28)	<0.001 (0.507)	1.67 (1.46)	3479/ 3135	1.17 (1.11–1.22)	<0.001 (0.131)	1.62 (1.46)	4806/ 4483	1.15 (1.10–1.20)	<0.001 (0.368)	1.57 (1.43)
CPAP use	1113/ 596	1.90 (1.72–2.10)	<0.001 (0.056)	3.21 (2.83)	1926/ 1196	1.68 (1.56–1.80)	<0.001 (0.001)	2.75 (2.49)	2557/ 1713	1.59 (1.43–1.69)	<0.001 (<0.001)	2.56 (2.21)
D10 vs. D20 (156,301 pairs)	D10/ D20				D10/ D20				D10/ D20			
OSA	1601/ 1563	1.04 (0.97–1.11)	0.311 (0.945)	1.24 (1.00)	3477/ 3402	1.06 (1.01–1.11)	0.022 (0.734)	1.31 (1.11)	4817/ 4844	1.05 (1.01–1.09)	0.026 (0.746)	1.28 (1.11)
CPAP use	1116/ 848	1.33 (1.22–1.46)	<0.001 (0.076)	1.99 (1.74)	1930/ 1574	1.26 (1.18–1.35)	<0.001 (0.263)	1.83 (1.64)	2572/ 2146	1.25 (1.18–1.33)	<0.001 (0.179)	1.81 (1.64)
D20 vs. S30 (353,106 pairs)	D20/ S30				D20/ S30				D20/ S30			
OSA	3873/ 3493	1.12 (1.07–1.18)	<0.001 (0.114)	1.49 (1.34)	9715/ 8853	1.12 (1.09–1.15)	<0.001 (0.199)	1.49 (1.40)	13,815/ 12,733	1.11 (1.08–1.14)	<0.001 (0.071)	1.46 (1.37)
CPAP use	2075/ 1472	1.42 (1.33–1.52)	<0.001 (0.006)	2.19 (1.99)	4326/ 3218	1.37 (1.31–1.43)	<0.001 (0.024)	2.08 (1.95)	5954/ 4544	1.34 (1.29–1.39)	<0.001 (0.003)	2.01 (1.90)
D20 vs. N100 (380,173 pairs)	D20/ N100				D20/ N100				D20/ N100			
OSA	4385/ 2966	1.49 (1.43–1.56)	<0.001 (0.085)	2.34 (2.21)	9713/ 6979	1.41 (1.36–1.45)	<0.001 (0.003)	2.17 (2.06)	13,811/ 9939	1.39 (1.36–1.43)	<0.001 (0.012)	2.13 (2.06)
CPAP use	2354/ 1290	1.84 (1.72–1.97)	<0.001 (0.002)	3.08 (2.83)	4325/ 2666	1.63 (1.56–1.72)	<0.001 (<0.001)	2.64 (2.49)	5951/ 3790	1.57 (1.50–1.63)	<0.001 (<0.001)	2.52 (2.37)
S30 vs. N100 (602,957 pairs)	S30/ N100				S30/ N100				S30/ N100			
OSA	6411/ 4999	1.28 (1.23–1.33)	<0.001 (0.013)	1.88 (1.76)	14,737/ 11,533	1.27 (1.24–1.30)	<0.001 (0.403)	1.86 (1.79)	21,074/ 16,444	1.26 (1.23–1.28)	<0.001 (0.262)	1.83 (1.76)
CPAP use	2680/ 2135	1.25 (1.18–1.33)	<0.001 (0.414)	1.81 (1.64)	5322 4454	1.18 (1.14–1.23)	<0.001 (0.018)	1.64 (1.54)	7586/ 6337	1.17 (1.13–1.21)	<0.001 (0.218)	1.62 (1.51)

Abbreviation: CI, confidence interval; CPAP, continuous positive airway pressure; OSA, obstructive sleep apnea; D10, severe vitamin D deficiency (serum 25(OH)D concentration ≤10 ng/mL); D20, mild vitamin D deficiency (11–20 ng/mL); S30, insufficient vitamin D status (21–30 ng/mL); N100, normal vitamin D status (31–100 ng/mL); P(p) for log rank test (proportionality test); E-value (lower 95% C.I.).

## References

[1] Mesquita J., Sola-Soler J., Fiz J.A., Morera J., Jane R. (2012). All night analysis of time interval between snores in subjects with sleep apnea hypopnea syndrome. Med. Biol. Eng. Comput..

[2] Mitra A.K., Bhuiyan A.R., Jones E.A. (2021). Association and Risk Factors for Obstructive Sleep Apnea and Cardiovascular Diseases: A Systematic Review. Diseases.

[3] Chiang J.K., Lin Y.C., Lu C.M., Kao Y.H. (2022). Snoring Index and Neck Circumference as Predictors of Adult Obstructive Sleep Apnea. Healthcare.

[4] Lam B., Ip M.S., Tench E., Ryan C.F. (2005). Craniofacial profile in Asian and white subjects with obstructive sleep apnoea. Thorax.

[5] Kapur V.K., Auckley D.H., Chowdhuri S., Kuhlmann D.C., Mehra R., Ramar K., Harrod C.G. (2017). Clinical Practice Guideline for Diagnostic Testing for Adult Obstructive Sleep Apnea: An American Academy of Sleep Medicine Clinical Practice Guideline. J. Clin. Sleep Med..

[6] Loh H.H., Lim Q.H., Kang W.H., Yee A., Yong M.C., Sukor N. (2023). Obstructive sleep apnea and vitamin D: An updated systematic review and meta-analysis. Hormones.

[7] Li X., He J., Yun J. (2020). The association between serum vitamin D and obstructive sleep apnea: An updated meta-analysis. Respir. Res..

[8] Kerley C.P., Hutchinson K., Bolger K., McGowan A., Faul J., Cormican L. (2016). Serum Vitamin D Is Significantly Inversely Associated with Disease Severity in Caucasian Adults with Obstructive Sleep Apnea Syndrome. Sleep.

[9] Bozkurt N.C., Cakal E., Sahin M., Ozkaya E.C., Firat H., Delibasi T. (2012). The relation of serum 25-hydroxyvitamin-D levels with severity of obstructive sleep apnea and glucose metabolism abnormalities. Endocrine.

[10] Bouloukaki I., Tsiligianni I., Mermigkis C., Bonsignore M.R., Markakis M., Pataka A., Steiropoulos P., Ermidou C., Alexaki I., Tzanakis N. (2021). Vitamin D deficiency in patients evaluated for obstructive sleep apnea: Is it associated with disease severity?. Sleep Breath..

[11] Archontogeorgis K., Voulgaris A., Chadia K., Bonelis K., Steiropoulos P. (2025). Effect of CPAP therapy on vitamin D status in patients with obstructive sleep apnea and chronic obstructive pulmonary disease overlap syndrome. Sleep Breath..

[12] Loh H.H., Sukor N. (2024). Obstructive sleep apnea and vitamin D level: Has the dust settled?. Clin. Respir. J..

[13] Liguori C., Romigi A., Izzi F., Mercuri N.B., Cordella A., Tarquini E., Giambrone M.P., Marciani M.G., Placidi F. (2015). Continuous Positive Airway Pressure Treatment Increases Serum Vitamin D Levels in Male Patients with Obstructive Sleep Apnea. J. Clin. Sleep Med..

[14] Siachpazidou D.I., Kotsiou O.S., Stavrou V., Pastaka C., Gogou E., Kechagia M., Varsamas C., Economou N.T., Zouridis S., Patrikioy E. (2021). Serum vitamin D levels in patients with obstructive sleep apnea syndrome and level changes after continuous positive airway pressure therapy. Sleep Breath..

[15] Liguori C., Izzi F., Mercuri N.B., Romigi A., Cordella A., Tarantino U., Placidi F. (2017). Vitamin D status of male OSAS patients improved after long-term CPAP treatment mainly in obese subjects. Sleep Med..

[16] Kulie T., Groff A., Redmer J., Hounshell J., Schrager S. (2009). Vitamin D: An evidence-based review. J. Am. Board. Fam. Med..

[17] Holick M.F. (2006). High prevalence of vitamin D inadequacy and implications for health. Mayo Clin. Proc..

[18] Crowe F.L., Jolly K., MacArthur C., Manaseki-Holland S., Gittoes N., Hewison M., Scragg R., Nirantharakumar K. (2019). Trends in the incidence of testing for vitamin D deficiency in primary care in the UK: A retrospective analysis of The Health Improvement Network (THIN), 2005–2015. BMJ Open.

[19] Dawson-Hughes B., Heaney R.P., Holick M.F., Lips P., Meunier P.J., Vieth R. (2005). Estimates of optimal vitamin D status. Osteoporos. Int..

[20] Holick M.F. (2007). Vitamin D deficiency. N. Engl. J. Med..

[21] Cui A., Zhang T., Xiao P., Fan Z., Wang H., Zhuang Y. (2023). Global and regional prevalence of vitamin D deficiency in population-based studies from 2000 to 2022: A pooled analysis of 7.9 million participants. Front. Nutr..

[22] Aranow C. (2011). Vitamin D and the immune system. J. Investig. Med..

[23] Puckrin R., Iqbal S., Zidulka A., Vasilevsky M., Barre P. (2015). Renoprotective effects of continuous positive airway pressure in chronic kidney disease patients with sleep apnea. Int. Urol. Nephrol..

[24] Nakashima A., Yokoyama K., Yokoo T., Urashima M. (2016). Role of vitamin D in diabetes mellitus and chronic kidney disease. World J. Diabetes.

[25] Upala S., Sanguankeo A. (2015). Association between 25-Hydroxyvitamin D and Obstructive Sleep Apnea: A Systematic Review and Meta-Analysis. J. Clin. Sleep Med..

[26] World Health Organization Ageing and Health. https://www.who.int/news-room/fact-sheets/detail/ageing-and-health.

[27] Moraes W., Piovezan R., Poyares D., Bittencourt L.R., Santos-Silva R., Tufik S. (2014). Effects of aging on sleep structure throughout adulthood: A population-based study. Sleep Med..

[28] Bixler E.O., Vgontzas A.N., Ten Have T., Tyson K., Kales A. (1998). Effects of age on sleep apnea in men: I. Prevalence and severity. Am. J. Respir. Crit. Care Med..

[29] Montserrat J.M., Ferrer M., Hernandez L., Farre R., Vilagut G., Navajas D., Badia J.R., Carrasco E., De Pablo J., Ballester E. (2001). Effectiveness of CPAP treatment in daytime function in sleep apnea syndrome: A randomized controlled study with an optimized placebo. Am. J. Respir. Crit. Care Med..

[30] Lips P. (2001). Vitamin D deficiency and secondary hyperparathyroidism in the elderly: Consequences for bone loss and fractures and therapeutic implications. Endocr. Rev..

[31] Holick M.F. (2024). Revisiting Vitamin D Guidelines: A Critical Appraisal of the Literature. Endocr. Pract..

[32] Hung K.C., Yu T.S., Lai Y.C., Hsu C.W., Yew M., Yu C.H., Chen I.W. (2025). Vitamin D deficiency and subsequent risk of obstructive sleep apnea: A multi-institutional retrospective study. Front. Nutr..

[33] Chuang M.H., Hsu W., Tsai Y.W., Hsu W.H., Wu J.Y., Liu T.H., Huang P.Y., Lai C.C. (2024). New-onset obstructive airway disease following COVID-19: A multicenter retrospective cohort study. BMC Med..

[34] Chalmers J.D., Polverino E., Crichton M.L., Ringshausen F.C., De Soyza A., Vendrell M., Burgel P.R., Haworth C.S., Loebinger M.R., Dimakou K. (2023). Bronchiectasis in Europe: Data on disease characteristics from the European Bronchiectasis registry (EMBARC). Lancet Respir. Med..

[35] Trepo E., Ouziel R., Pradat P., Momozawa Y., Quertinmont E., Gervy C., Gustot T., Degre D., Vercruysse V., Deltenre P. (2013). Marked 25-hydroxyvitamin D deficiency is associated with poor prognosis in patients with alcoholic liver disease. J. Hepatol..

[36] Vogiatzi M.G., Jacobson-Dickman E., DeBoer M.D., for the Drugs, and Therapeutics Committee of The Pediatric Endocrine Society (2014). Vitamin D supplementation and risk of toxicity in pediatrics: A review of current literature. J. Clin. Endocrinol. Metab..

[37] Dawson-Hughes B., Mithal A., Bonjour J.P., Boonen S., Burckhardt P., Fuleihan G.E., Josse R.G., Lips P., Morales-Torres J., Yoshimura N. (2010). IOF position statement: Vitamin D recommendations for older adults. Osteoporos. Int..

[38] Nair S. (2010). Vitamin d deficiency and liver disease. Gastroenterol. Hepatol..

[39] Williams S., Malatesta K., Norris K. (2009). Vitamin D and chronic kidney disease. Ethn. Dis..

[40] Hemmelmann C., El Galta R., Wang J., Schmitt S., Arani R. (2025). Implementation of the Anchor-Based Indirect Comparison Method for Equivalence Margin Derivation in Biosimilar Development. Pharmaceuticals.

[41] Sanchez-de-la-Torre M., Gracia-Lavedan E., Benitez I.D., Sanchez-de-la-Torre A., Moncusi-Moix A., Torres G., Loffler K., Woodman R., Adams R., Labarca G. (2023). Adherence to CPAP Treatment and the Risk of Recurrent Cardiovascular Events: A Meta-Analysis. JAMA.

[42] Theorell-Haglow J., Hoyos C.M., Phillips C.L., Yee B.J., Herrmann M., Brennan-Speranza T.C., Grunstein R.R., Liu P.Y. (2018). Changes of vitamin D levels and bone turnover markers after CPAP therapy: A randomized sham-controlled trial. J. Sleep Res..

[43] Rotenberg B.W., Murariu D., Pang K.P. (2016). Trends in CPAP adherence over twenty years of data collection: A flattened curve. J. Otolaryngol. Head Neck Surg..

[44] Yao N., Ma C., Dou R., Shen C., Yuan Y., Li W., Qu J. (2024). Exploring the link between vitamin D deficiency and obstructive sleep apnea: A comprehensive review. J. Sleep Res..

[45] Ke L., Graubard B.I., Albanes D., Fraser D.R., Weinstein S.J., Virtamo J., Brock K.E. (2013). Hypertension, pulse, and other cardiovascular risk factors and vitamin D status in Finnish men. Am. J. Hypertens..

[46] Scragg R.K., Camargo C.A., Simpson R.U. (2010). Relation of serum 25-hydroxyvitamin D to heart rate and cardiac work (from the National Health and Nutrition Examination Surveys). Am. J. Cardiol..

[47] Giustina A., Bouillon R., Dawson-Hughes B., Ebeling P.R., Lazaretti-Castro M., Lips P., Marcocci C., Bilezikian J.P. (2023). Vitamin D in the older population: A consensus statement. Endocrine.

[48] Benjafield A.V., Ayas N.T., Eastwood P.R., Heinzer R., Ip M.S.M., Morrell M.J., Nunez C.M., Patel S.R., Penzel T., Pepin J.L. (2019). Estimation of the global prevalence and burden of obstructive sleep apnoea: A literature-based analysis. Lancet Respir. Med..

